# The Effect of Intravenous Magnesium Sulphate as an Adjuvant in the Treatment of Acute Exacerbations of COPD in the Emergency Department: A Double-Blind Randomized Clinical Trial

**DOI:** 10.4314/ejhs.v31i2.9

**Published:** 2021-03

**Authors:** Fatemeh Jahanian, Iraj Goli Khatir, Hamed Amini Ahidashti, Sepideh Amirifard

**Affiliations:** 1 Department of Emergency Medicine, Gut & Liver Research Center, Mazandaran University of Medical Sciences, Sari, Iran; 2 Department of Emergency Medicine, Diabetes Research Center, Mazandaran University of Medical Sciences, Sari, Iran

**Keywords:** Chronic Obstructive Pulmonary Disease, COPD exacerbation, Sulfate, Magnesium, Controlled Clinical Tria

## Abstract

**Background:**

Acute exacerbations of chronic obstructive pulmonary disease (AECOPD) are serious complications that often require immediate intervention in an emergency department (ED). The aim of this study was to investigate the effect of intravenous magnesium sulphate as an adjuvant in the treatment of AECOPD in the ED.

**Methods:**

In a double-blind, randomized clinical trial, a total of 60 patients with AECOPD presenting to the ED of Imam Khomeini Hospital in Sari, Iran, were included. The study was conducted between September 2016 and February 2018. Eligible patients were randomly allocated into two groups of intervention and control. Patients in the intervention and control groups received intravenous infusion of magnesium sulfate (2 gr) or normal saline over 30 minutes, respectively. For all patients, Borgdyspnea score, forced expiratory volume in one second (FEV1) result and clinical variables of interest were evaluated before the beginning of the intervention, and also 45 minutes and 6 hours after the commencement of intervention.

**Results:**

Regardless of time of evaluation, pulse rate (PR), respiratory rate (RR) and Borg score in intervention group was lower than control group. Also, FEV1 and SPO2 were greater in intervention group compared to control group. However, these differences were not statistically significant (between-subject differences or group effect) (p<0.001). The trends of FEV1, SPO2, PR, RR and Borg score were similar between two groups of study (no interaction effect; P>0.05).

**Conclusion:**

According to the results of this study, it seems that using intravenous magnesium sulfate has no significant effect on SPO2, FEV1, RR, and PR of patients with AECOPD who presented to ED.

## Introduction

Chronic Obstructive Pulmonary Disease (COPD) is one of the leading causes of hospitalization, mortality, and increased consumption of medical expenses (1–2). In the natural course of disease, patients with COPD may experience a severe acute exacerbation of the disease. Acute exacerbation of COPD (AECOPD) is a serious complication that often requires immediate intervention in an emergency department (ED) (3). AECOPD is usually associated with a significant worsening lung function, accelerated decline in FEV1, and may rapidly progress to acute respiratory failure which is life-threatening and causes increased short-term mortality and morbidity (4–5). Therefore, appropriate, effective and timely management of COPD patients who suffer from acute exacerbation in the course of the disease is necessary. Despite substantial advances in the pharmacologic and non-pharmacologic treatments for AECOPD, no progress has been made in treatment over the past decades, and most treatments are only partly effective. Therefore, the optimal management of AECOPD is an urgent research priority (4–7).

Bronchodilators, corticosteroids, oxygen and antibiotics are the standard treatment for AECOPD. Recently, intravenous magnesium sulfate has been suggested as a potential adjunct therapy for AECOPD. It has been previously shown that magnesium can potentiate the bronchodilatory effect of inhaled beta-2 agonist and has beneficial effects on the function of respiratory muscle (6–9). In addition, due to its anti-inflammatory effects, it can theoretically potentiate resolution of acute airway inflammation and AECOPD (9–11). Several studies evaluated the effect of intravenous or inhalation magnesium sulfate for treatment of AECOPD, with conflicting results (2 6,10–11). Although some previous studies supporting the efficacy of magnesium sulphate in AECOPD, evidences are relatively weak and inconsistent. Therefore more studies with robust design are needed to evaluate its efficacy (5, 11–14).

Therefore, considering the paucity of information and conflicting available evidence, the aim of this study was to evaluate the effect of intravenous magnesium sulphate as an adjuvant in the treatment of AECOPD in the ED.

## Methods

**Study design and sample**: In a double-blind, randomized clinical trial, a total of 60 patients with AECOPD presenting to the ED of Imam Khomeini Hospital in Sari, Mazandaran Province, Iran, were included. The study was conducted between September 2016 and February 2018.

**Inclusion and exclusion criteria**: The inclusion criteria were known clinically diagnosed moderate COPD (FEV1/FVC<70%, 30<FEV1<50, often symptomatic with shortness of breath and sometimes with the acute attack symptoms including changes in the severity of shortness of breath, cough, and sputum volume) and not having diseases that mimic the clinical features of COPD. Patients who required tracheal intubation or noninvasive ventilation on admission to ED, having a history of cardiac disease, not able to cooperate or undergo spirometry and with reduced level of consciousness were excluded from the study. Others exclusion criteria were use of intravenous magnesium sulfate in the past 24 hours, systolic blood pressure <100mmHg, using medications that contain magnesium, presence of hyperkalemia or hypocalcaemia with ECG abnormalities, respiratory rate <16 and using magnesium hydroxide oraluminum-magnesium hydroxide syrups during the past 24 hours.

**Data collection**: Eligible patients were randomly divided into two groups of intervention and control with equal sample size. Randomization was done using a sealed envelope technique with a computer-generated random numbering system with the help of a nurse who was blinded to the study groups. In addition to conventional standard therapy which includes nasal-cannula oxygen therapy (2–3 L/min), inhaled salbutamol and ipratropium (6 puff every 20 minutes until three times), and also intravenous hydrocortisone (100 mg), patients in the intervention and control groups received intravenous infusion of magnesium sulfate (2 gr in 100 ml of normal saline) or normal saline (100 ml) over 30 minutes, respectively. In both groups, the study was conducted after receiving standard treatment for COPD exacerbation, within the first 60 minutes and the patients were clinically stabilized. The doses of medications were the same for all patients. All drugs were prepared in syringes in the same size, color, volume, and shape. The syringes were labeled as A or B by a pharmacist and were administered by a nurse, using a peripheral venous catheter. Also, patients were unaware of the type of medication they were receiving.

Throughout the study, patients were monitored for possible side effects. Fifteen minutes after the start of the magnesium sulfate infusion, patellar reflex was assessed in patients, and if the patella reflex was diminished or absent, magnesium sulfate was discontinued immediately.

Before the beginning of the intervention (T1), and also 45 minutes (T2) and 6 hours after the commencement of intervention (T3), spirometry was performed for all patients (for evaluating FEV1). Also, the respiratory rate (RR), pulse rate (PR), O2 saturation (SPO2), presence of cyanosis, and 0–10 Borg dyspnoea scale (0 indicate the absence of dyspepsia and 10 indicates severe dyspnea) were evaluated.

**Ethical consideration**: The present study was conducted after the approval of the institutional ethics committee. The researchers explained the objectives of the study to the participants, and informed consent was obtained from all participants. Also, this study was registered in the Iranian Registry of Clinical Trials Database (IRCT20150315021480N8).

**Sample size**: A priori sample size was calculated using GPower3.1 with the formula for calculation of samples of repeated measures, based on a presumed effect size of 0.2, a statistical power of 80%, and a type I error of 5%. The overall proper sample size was found to be 54 participants. We therefore recruited 60 patients to account for any dropouts.

**Statistical analysis**: Shapiro-Wilk test was used to evaluate whether data were normally distributed. Descriptive baseline characteristics for two groups were tabulated as Mean (SD), or percentages. The independent t-test was used to compare quantitative variables and the chi-square test was used to compare qualitative variables. Using General Linear Model (GLM), FEV1, RR, PR, SPO2 and Borg dyspnoea score were compared between two groups by repeated measurement ANOVA test. Time of evaluation was considered as within subject factor, intervention state (intravenous magnesium sulfate and control) as between subject factor. Data were performed using the SPSS software (version 20.0, SPSS Inc., Chicago, IL, USA). For all statistical tests, P value <0.05 was considered as statistically significant.

**Data sharing**: All relevant data and methodological detail pertaining to this study are available to any interested researchers upon reasonable request to the corresponding author.

## Results

**Figure 1:**
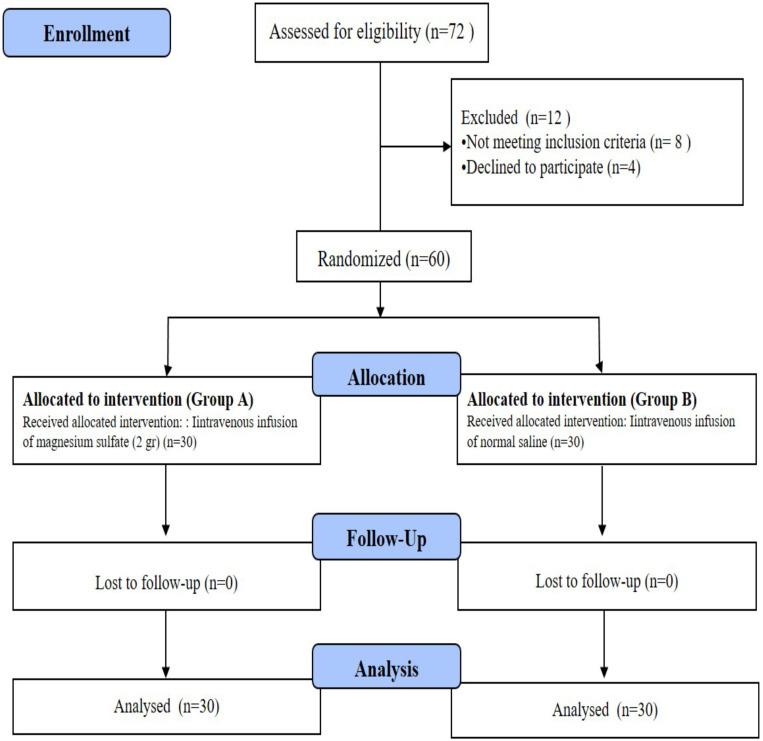
Flow chart of the study

During the study period, 72 consecutive patients with AECOPD presenting to the ED were screened. Eight patients did not meet the inclusion criteria and four patients declined to participate in the study. All the remaining 60 patients were randomly allocated to two groups and completed the study (Figure 1).

In the intervention and control groups, 83.3% (n=25) and 70% (n=21) of the patients were females, respectively (P=0.635). The mean age of the patients in intervention and control groups was 62.9±7.3 and 65.8±3.1 years, respectively (P=0.435). 53.3% and 56.7% of patients in intervention and control groups were current cigarettes smoker, respectively (P=0.92). Most of the patients in intervention (n=20; 67%) and control groups (n=18; 60%) had disease duration <5 years, respectively (P=0.75). Dyspnea was the most common complaint of patients (55% of all patients). Ninety-three percent (n=28) of patients in the intervention group and 90% (n=27) in the control group were concurrently on medical treatment (P=0.86).

Table 1 shows the mean and SD values of the FEV1, Borg scores, RR, PR and SPO2 of each group. There was no significant differences between the groups at the beginning of the study(P>0.05). As shown in Table 1, there was a statistically significant time trend (within-subject differences or time effect) regardless of group of study (p<0.001). Regardless of time of study, PR, RR and Borg score in intervention group (magnesium sulfate) were lower than control group. Also, FEV1 and SPO2 was greater in intervention group compared to control group. However, these differences were not statistically significant (between-subject differences or group effect) (p<0.001). The trends of FEV1, SPO2, PR, RR and Borg score were similar between two groups of study (no interaction effect; P>0.05). Also, in both groups, patients did not have cyanosis at the beginning and during the study period.

**Table 1:** Mean (SD) of FEV1, Borg score, RR, RR and SPO2 at before and after intervention times in both groups

Variable		Time			*P*-value*		
		
		T1	T2	T3	Time effect	Group effect	Interaction effect
FEV1	Intervention	52.56 (6.66)	58.06 (5.75)	61.66(5.34)	<0.001	0.23	0.78
Mean(SD)	Control	50.90 (7.8)	55.96 (6.08)	59.83 (5.81)			
	*P*-value**	0.20	0.53	0.28			
**Borg**	Intervention	8.16(0.59)	6.33 (0.84)	4.73 (1.04)	<0.001	0.89	0.73
**score**	Control	8.20 (0.61)	6.43 (0/89)	4.86 (0.93)			
Mean(SD)	*P*-value**	0.71	0.56	0.79			
**RR**	Intervention	31.26(4.56)	25.40 (3.82)	21.20 (2.85)	<0.001	0.22	0.15
Mean	Control	33.06(5.21)	26.73 (3.94)	21.60(3.16)			
(SD)	*P*-value**	013	0.70	0.33			
**PR**	Intervention	91.30(10.27)	81.56(7.33)	79.26(5.18)	<0.001	0.67	0.79
Mean(	Control	92.46 (9.43)	82.36 (7.67)	79.60 (4.49)			
SD)	*P*-value**	0.66	0.35	0.97			
**SPO2**	Intervention	91.33(1.68)	94 (1.98)	95.23 (2.35)	<0.001	0.66	0.89
Mean	Control	91.16(1.85)	93.70 (2.05)	95.06 (2.47)			
(SD)	*P*-value**	0.43	0.70	0.67			

T1: Before the beginning of the intervention; T2: 45 minutes after the commencement of intervention; T3: 6 hours after the commencement of intervention

FEV1: Forced expiratory volume in one second; RR: Respiratory rate; PR: Pulse rate; SPO2: Oxygen saturation

* Repeated measurement ANOVA

** Independent Samples t Test

## Discussion

The present study showed that although intravenous magnesium sulfate improves FEV1 and SPO2, and decreases PR, RR and Borg dyspnea score, compared to placebo, in patients with AECOPD in the ED; the differences were not statically significant. Based on previous evidence, the efficacy of intravenous magnesium sulfate in patients with exacerbated asthma has been confirmed (15–17). Magnesium can potentially inhibit the contraction of bronchial smooth muscle (18). In addition, it has been shown that hypomagnesaemia can cause hyperactive airways (19,20). To add to this, several studies have shown that intravenous magnesium sulfate improves bronchospasm and dyspnea in asthmatic patients (17, 21–22). On the other hand, another study showed that intravenous magnesium sulfate is effective only in children and has no effect on adults with acute asthma. However, due to the acceptable safety profile of magnesium sulfate, its use in adults with life-threatening conditions was recommended (23). Also, a recent meta-analysis confirmed the efficacy of intravenous magnesium sulfate in reducing the need of hospitalization and improving recovery in children with acute asthma (24). Meanwhile, despite the positive effect of magnesium sulfate in asthma, less information is available regarding the use of magnesium sulfate in AECOPD (11). In line with the results of our study, Nouira et al. (14) showed that although intravenous bolus of magnesium sulfate improved the dyspnea score and peak expiratory flow rate (PEFR) in AECOPD patients, the differences were not statistically significant. In another study, it was shown that using 2 gr of intravenous magnesium sulfate does not have any significant effect on FEV1 and PEER in patients with AECOPD (2). Edwards et al. revealed that in patients with AECOPD, using nebulized magnesium in combination with salbutamol, has no significant effect on FEV1 (10). Vafadar Moradi et al. (9) showed that intravenous administration of 2.5 gr of magnesium sulfate significantly reduced the RR, PEFR and dyspnea severity scorein patients with AECOPD. However, in terms of SPO2, no significant differences was observed. The results of this study are somewhat inconsistent with our findings. Additionally, two other studies showed a significant increase in FEV1 after administration of magnesium sulfate, in patients with AECOPD (6–7). It is believed that although intravenous magnesium sulfate did not have an immediate effect on FEV1, it enhances the effect of beta 2 agonist bronchodilators (22). However, no such comparison was made in the present study. Also, previous evidences indicate a conflicting data regarding the correlation between serum magnesium levels and clinical features in patients with COPD (25–27). Using different doses of magnesium and having different inclusion and exclusion criteria, such as considering critically ill patients or excluding them from the study, can be possible explanations for this difference. During this study, no significant adverse effects were identified. Although magnesium sulfate appears to be generally safe, high doses of this drug can cause serious cardiovascular reactions and respiratory depression (28). This study has some limitations. We did not evaluate the magnesium serum level. Also, this is a single-center study that may limit the generalizability of the study. In conclusion, according to the results of this study, it seems that using intravenous magnesium sulfate has no significant effect on SPO2, FEV1, RR and PR of patients with AECOPD who presented to ED. However, further well-designed clinical trials are warranted to evaluate the efficacy and determine the optimal doses and route of administration of magnesium sulfate in patients with AECOPD.
